# EGFR and COX-2 protein expression in non-small cell lung cancer and the correlation with clinical features

**DOI:** 10.1186/1756-9966-30-27

**Published:** 2011-03-07

**Authors:** Feng Li, Yongmei Liu, Huijiao Chen, Dianying Liao, Yali Shen, Feng Xu, Jin Wang

**Affiliations:** 1 Radiation Oncology, Tumor Center, West China Hospital, Sichuan University, China; 2 Department of Pathology, West China Hospital, Sichuan University, China

## Abstract

**Background:**

To evaluate the expression of EGFR and COX-2 and their correlation with prognosis in NSCLC

**Methods:**

The paraffin embedded tumor samples of 50 NSCLC patients receiving radical resection were analyzed immunohistochemically for EGFR and COX-2 expression and their prognostic values were explored.

**Results:**

The positive rate of EGFR protein in NSCLC tumor cells was 46%, which was significantly higher than its expression in normal lung (p = 0.0234) and paracancerous tissues (p = 0.020). EGFR expression was significantly higher in nodal positive than in nodal negative patients (p = 0.04). The mean survival time for EGFR positive patients (31 months) was significantly lower than that for patients with EGFR negative expression (48 months) (p = 0.008,). In patients receiving post-operation thoracic irradiation, the mean survival time for EGFR positive patients was significantly lower than that for patients without EGFR positive expression (25 vs. 48 months, P = 0.004). The positive rate of COX-2 protein expression in NSCLC tumor cells was 90%, which was significantly higher than that in normal tissue(p = 0.00) and paracancerous tissue (p = 0.00). There was no correlation between COX-2 expression and patient survival, and no correlation between COX-2 and EGFR protein expression (P = 0.555).

**Conclusions:**

COX-2 and EGFR are over-expressed in NSCLC. EGFR is an independent prognostic factor and a predictive factor for radiotherapy response in NSCLC.

## Background

Lung cancer is the leading cause of death world wide. The non-small cell lung cancer (NSCLC) accounts for 75-85% among all lung cancers. The conventional treatment e.g. surgery, radiotherapy and chemotherapy yields a dismal overall 5-year survival of 14% which necessitates the development of new treatment options [1]. With advances in cytogenetic and molecular biology, the detection and analysis of tumor suppressor gene and oncogene may provide predictive values for prognosis and treatment choice for NSCLC. Among these molecular markers, the epidermal growth factor receptor (EGFR) and cyclooxygenase-2 (COX-2) over expression are common in NSCLC [2-9].

EGFR (HER1, ErbB) is a transmembrane glycoprotein with three functional domains: an extracellular domain containing two EGF binding sites; a hydrophobic transmembrane domain and a cytoplasmic domain (tyrosine kinase (TK) and a carboxyl autophosphorylation region) [10,11]. EGFR is abnormally upregulated and activated in a variety of tumors [12]. Deregulation of receptor tyrosine kinases as a result of overexpression or activating mutations leads to the promotion of cell proliferation or migration, inhibition of cell death, or the induction of angiogenesis [13,14].

The expression and activity of EGFR are determinants of response to target therapy and radiosensitivity in several tumour types [15]. EGFR overexpression in non-small cell lung cancer (NSCLC) is variable ranging from 19% to 89% and its prognostic value remains controversial [16,17]. COX-2 over expression is also found in many tumor types [18]. The carcinogenic effect of COX-2 mainly exerted through the increase of prostaglandin levels (PGE2, PGF2a, PGD2, TXA2, PGI2 and PGJ2). In lung cancer, COX-2 expression has been reported to inhibit apoptosis [19], promote angiogenesis [20] and metastasis [2]. It has been reported in a recent meta-analysis that COX-2 might be an independent prognostic factor for NSCLC [21]. COX-2 inhibitor has been investigated in both pre-clinical and clinical study, and has shown synergistic effects with radiation and chemtoxic drugs on tumor [3,22]. COX-2 catalyzes the conversion of arachidonic acid into prostanoids including prostaglandin E2, which is often associated with oncogenesis of lung tumors. The oncogenic signals are transducted through the MAPK/Erk pathway [23] which therefore closely correlates EGFR with COX-2. A number of *in vitro *studies have postulated a link between EGFR activation and subsequent COX-2 upregulation. The relationship between these factors has not been established in patients with NSCLC.

In order to evaluate the EGFR and COX-2 expression and their impact on prognosis of NSCLC patients receiving post-operative adjuvant therapy, the paraffin embedded tumor samples from 50 NSCLC were analyzed immunohistochemically for EGFR and COX-2 expression and their prognostic values were explored.

## Methods

### Tumor specimen

Paraffin-embedded tissue sections from 50 histopathologically proven NSCLC patients who received radical resection during June 2001 and March 2004 were collected.

### Patient data

All patients were histopathologically diagnosed NSCLC and had not received preoperative chemotherapy nor radiotherapy. Among them there were 31 males and 19 females, aged 36-76 (mean 58) years. According to WHO classification (2000), there were 21 squamous, 26 adenomatous and 3 adenosquamous carcinomas, with 40 moderate and well differentiated (G1-G2) and 10 low differentiated (G3). 15 cases were staged I-II and 35 III-IV based on the revised AJC staging for lung cancer (1997). Thirty-nine cases had intra-thoracic lymph node metastasis (N1-N2), and 11 were negative lymph node metastasis. The paracancerous tissues (defined as more than 5 cm away from the carcinoma tissue) taken from 7 cases and the normal tissues from 6 cases were used as controls. All patients received 4 cycles of adjuvant platinum based two drug chemotherapy. Among them, 28 patients received post-operative combined chemotherapy and thoracic radiotherapy and 22 patients had chemotherapy alone.

### Immunohistochemistry (IHC)

The paraffin embedded tumor specimens were cut into 4-um sections for IHC staining against EGFR and COX-2 according to the manufacturer's instructions. In brief, after deparaffinization and rehydration, the samples were treated with sodium citrate buffer and microwave for epitope retrieval, block non-specificity antigen with normal goat serum incubating 10 minutes; After a washing procedure with distilled water, tissue sections were covered for 5 min with 3% H_2_O_2 _to block endogenous peroxidase, followed by an additional washing procedure with the supplied buffer. Slides were then placed in a 37°C water bath and incubated for 30 min with the primary mouse anti-EGFR MAb (Chemicon International, Inc.) diluted 1:200 and anti-COX-2 MAb (Beijing Zhongsan Biological Company) diluted 1:100. After two rinses in buffer the slides were incubated with the detection system for 30 min. Tissue staining was visualized with a DAB substrate chromogen solution. Slides were counterstained with hematoxylin, dehydrated, and mounted. To validate each staining, the EGFR positive colon cancer section provided with the EGFR kit was used as positive control in each staining run. For COX-2 staining, the positive control used the sample itself (internal control). The negative control for both EGFR and COX-2 used PBS to substitute the primary antibody.

### Scoring method

The EGFR positive cell is defined as having clearly shown brownish yellow granules within cytoplasm and cell membrane; the COX-2 positive cell having clearly shown brown granules in cytoplasm; with clear background. Slide evaluation was independently performed by two investigators blinded to all subject characteristics. The slides were first observed for staining status under low power microscope, and then randomly selected 5 fields under high power (200×) light microscope. For assessment of staining positivity, the number of positive cells out of 200 tumor cells in each field was counted. The positive cell counts from all 5 fields were averaged and then divided by the total cell number of 5 fields to get the positivity ratio. Staining positivity was defined if the ratio ≥ 10% (+), and negative if ration < 10% (-). As EGFR and COX-2 were not expressed in normal tissues, any observed positivity of EGFR and COX-2 was thus considered as over expression [4].

### Statistical analysis

The data were analyzed using SPSS 13.0 software package. The correlation of EGFR expression with different clinical characteristics was analyzed with chi-square test. COX proportional-hazards model was used to analyze the correlation of survival with various clinical characteristics and EGFR protein expression. The Kaplan-Meier method and Log-rank test were used to analyze the correlation of patient survival with EGFR expression. A significance level of P < 0.05 was used.

## Results

### EGFR protein expression

**Figure 1:**
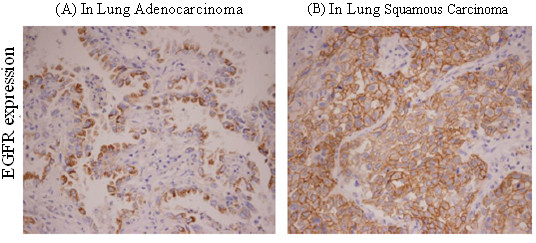
**EGFR protein expression in (A) adenocarcinoma and (B) squamous carcinoma of the lung by immunohistochemical assay (×200)**.

The positive rate of EGFR protein in NSCLC tumor cells were 46%, which was significantly higher than its expression in normal lung (*p *= 0.0234) and paracancerous (*p *= 0.020)(Figures 1A &1B, Tables 1 &2).

**Table 1:** Comparing EGFR protein expression in neoplastic and normal tissue

Tissue type	Number of cases	EGFR		Positive rate(%)	P value
				
		positive	negative		
Neoplastic tissue	50	23	27	46	0.034*
Normal tissue	6	0	6	0	

*p < 0.05

**Table 2:** Comparing EGFR protein expression in neoplastic and paracancerous tissue

Tissue type	Number of cases	EGFR		Positive rate(%)	P value
				
		positive	negative		
Neoplastic tissue	50	23	27	46	0.020*
Paracancerous tissue	7	0	7	0	

*p < 0.05

### Correlation between EGFR expression and clinical features

The expression of EGFR in different subgroups were compared and summarized in Table 3. It shows that the difference of EGFR expression was only significant between the nodal positive and negative subgroups (56.4% vs.10%, p = 0.04). There is no significant difference between age (60 vs. under 60 ys), gender, adeno- vs. non-adenocarcinoma, the differentiation of tumor, and staging.

**Table 3:** EGFR expression and clinical characteristics

Clinical features	EGFR		Positive expression rate	*P *value
			
	positive	negative		
Ages				0.448
≤60	18	14	43.80%	
>60	9	9	50%	
Sex				0.445
Male	16	15	40.50%	
Female	11	8	42.10%	
Pathologic type				0.543
Squamous carcinoma	13	8	40%	
Adencarcinoma	13	13	50%	
Mixed type	1	2	66.70%	
Tumor length				0.827
≤3 cm	9	7	43.80%	
>3 cm	18	16	47.10%	
Level of Differentiation				0.474
Poor Differentiated	6	4	40%	
Moderate and Well Differentiated	21	19	47.50%	
TNM Stage				0.129
I-II	11	5	40%	
III	13	15	50.60%	
IV	3	3	50%	
Lymph node				0.006*
N0	9	1	10%	
N1-3	17	22	56.40%	

*p < 0.05

### EGFR expression and overall survival

**Figure 2:**
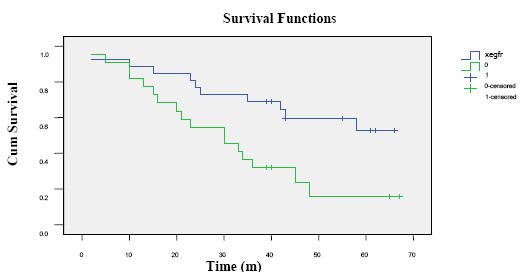
**Survival curves with different level of EGFR protein expression**. The solid blue line indicates the survival for EGFR negative and the green line represents survival for EGFR positive expression subgroups.

Cox proportional hazards analysis showed that EGFR protein positive expression independently predicted patient survival, with RR of 2.311, *p *= 0.038, and 95% confidence interval (CI) of 1.049 - 5.095. The mean survival time for EGFR positive patients was 31 months, whereas the survival time was 48 months for patients with EGFR negative expression, with the latter significantly longer than the former (*p *= 0.008, Log Rank (Mantel-Cox))(Figure 2).

### EGFR expression and outcome of radiotherapy

**Figure 3:**
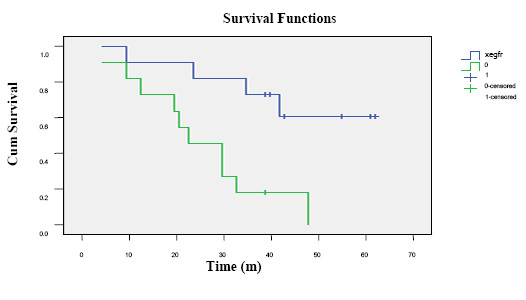
**Survival curves based on EGFR expression in patients receiving thoracic irradiation**. The solid blue line indicates the survival for EGFR negative and the green line represents survival for EGFR positive expression subgroups.

In patients receiving post-operation thoracic irradiation, the mean survival time for EGFR positive patients (n = 15)was 25 months which was significantly shorter than that (48 months)for patients (n = 13)with EGFR negative expression (P = 0.004)(Figure 3).

### COX-2 expression

**Figure 4:**
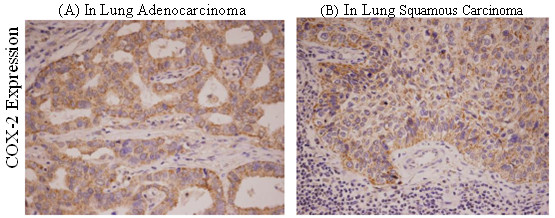
**Immunohistochemical stain(×200)for COX-2 expression in (A) adenocarcinoma and (B) squamous carcinoma of the lung**.

The positive rate of COX-2 protein expression in NSCLC tumor cells was 90%, which was significantly higher than that in normal tissue(p = 0.00) and paracancerous tissue (p = 0.00)(Figure 4, Tables 4 and 5).

**Table 4:** COX-2 expression in neoplastic and normal tissue

Tissue type	Number of cases	COX-2		Positive rate(%)	P value
				
		positive	negative		
Neoplastic tissue	50	40	5	90	0.000*
Normal tissue	6	0	6	0	

*p < 0.05

**Table 5:** COX-2 expression in tumor and paracancerous tissue

Tissue type	Number of cases	EGFR		Positive rate(%)	P value
				
		positive	negative		
Neoplastic tissue	50	40	5	90	0.000*
Paracancerous tissue	7	1	6	14.3	

*p < 0.05

The COX-2 expression was 100% in adenocarcinoma and significantly higher than that in squamous carcinoma (76.2%) of the lung. No correlation was found between COX-2 expression and patient survival (Figures 4, Table 6).

**Table 6:** COX-2 expression and correlation with clinical features

Clinical features	EGFR		Positive expression rate	*P *value
			
	-	+		
Ages				0.599
≤60	3	30	90.90%	
>60	2	15	88.20%	
Sex				0.362
Male	4	27	87.10%	
Female	1	18	94.70%	
Pathologic type				0.022*
Squamous carcinoma	5	16	76.20%	
Adencarcinoma	0	26	100%	
Mixed type	0	3	100%	
Tumor length				0.518
≤3 cm	2	14	87.50%	
>3 cm	3	31	91.20%	
Level of Differentiation				0.258
Poor Differentiated	2	8	80%	
Moderate and Well Differentiated	3	37	92.50%	
TNM Stage				0.129
I-II	11	5	40%	
III	13	15	50.60%	
IV	3	3	50%	
Lymph node				0.006*
N0	9	1	10%	
N1-3	17	22	56.40%	

*p < 0.05

### EGFR and COX-2 expression on chemotherapy outcome

Based on COX proportional hazards analysis which also takes account of other clinical characteristics, there was no correlation of EGFR and COX-2 expression with overall survival in 22 patients receiving chemotherapy alone (P > 0.05).

### Correlation of EGFR and COX-2 expression

As shown in Table 7, no correlation was found between COX-2 and EGFR protein expression (Χ2 = 0.112, P = 0.555).

**Table 7:** Correlation of EGFR and COX-2 protein expression

		EGFR		Total
			
		negative	positive	
COX-2	negative	3	2	5
	positive	25	23	48
Total		28	25	53

There was no significant relationship between COX-2 and EGFR. Χ2 = 0.112, P = 0.555.

## Discussion

EGFR and COX-2 are molecular targets which have shown importance for NSCLC. Previous studies reported that the levels of EGFR and COX-2 expression might correlate with poor disease prognosis and reduced survival [20,24]. In this study the prognostic values of EGFR and COX-2 were evaluated with immunohistochemical assay.

Activation of the EGFR results in activation of downstream signaling pathways, including the Ras-Raf-MKK-extracellular signal-regulated kinase (ERK) and lipid kinase phosphatidylinositol 3-kinase/Akt pathways. Dysregulation of these pathways can result in oncogenesis and cancer progression [4,25-27]. Similarly, our results implied that EGFR over-expression participated in lung cancer development. EGFR expression was negative in paracancerous and normal tissues, which was significantly lower than that in lung cancer tissue (46%)(*P < 0.05*). It was similarly reported in studies with the utilization of the immunohistochemical assay that EGFR expression was very low in normal tissue but often over-expressed in lung cancer tissue. In normal tissue, EGFR expression was limited to the basal layer of the epithelium where proliferation occured. EGFR expression was significantly increased in dysplastic cells, indicating that EGFR pathway involves in lung cancer development [28]. Therefore, the detection of EGFR expression in tissue sample before surgery might be helpful in diagnosis of NSCLC.

In our study EGFR expression in NSCLC was not significantly correlated with patients' age, gender, histopathologic type, cell differentiation, tumor size and TNM stages (P > 0.05). However, EGFR over-expression was correlated with lymph node metastasis, the probability of lymph node metastasis was significantly greater in patients with EGFR over-expression than in EGFR negative group (*P = 0.006*). This might indicate that EGFR was not only involved in cancer genesis but also played an important role in cancer progression. Though EGFR was most commonly found in squamous cell (70%) followed by adenocarcinoma (50%) [29], and large cell carcinomas [28], in our study, EGFR positivity rates were similar between squamous carcinoma (40%) and adenocarcinorma (50%). This discrepancy might be explained by the small sample size of our study which could limit the power of detection.

Our results showed that EGFR positive expression was an independent prognostic factor for NSCLC, among various factors including patient's age, gender, histopathology, tumor differentiation, tumor size, TNM staging and chemotherapy/radiotherapy. Based on the COX proportional hazard analysis adjusting for other significant variables, the mortality of patients with positive tumor EGFR expression was 2.31 times that of the EGFR negative NSCLC (*P < 0.05*). Nicholson *et al *[30] reported a meta-analysis based on 200 studies published in Medline between 1985 and 2000, which showed that EGFR over-expression was correlated with patient's prognosis in 10 tumor types. But only 30% of the studies considered EGFR to be associated with NSCLC prognosis. However, it might not be conclusive since some of the studies in the meta-analysis did not include treatment for multivariate analysis, which might have an impact on survival.

A recent study reported that EGFR positive expression assessed by IHC in NSCLC was associated with better survival in patients receiving EGFR TKI [31], which was contrasted to our study that EGFR positivity predicted for worse survival in patients treated with radiotherapy. In our study, for patients receiving radiotherapy, the mean survival for EGFR positive patients (25 months) was significantly lower than that for EGFR non-positive patients (48 months) (*p *= 0.004). It suggested that EGFR positivity might relate to resistance to radiotherapy, which agreed with the finding from head and neck study that EGFR expression was correlated with radiation resistance [32]. However, no such findings have been reported in NSCLC, and further prospective studies with larger sample size are needed to validate the role of EGFR in NSCLC response to radiation. To better evaluate the prognostic value of EGFR in NSCLC, the detection of activated EGFR (e.g. EGFR phosphorylation) or combined detection with other molecular markers should be used [33].

In our study the positive rate of COX-2 protein expression was 90% for NSCLC tumors and was significantly higher than that for normal lung (0%) and paracancerous tissue (14.3%). Therefore, it suggested that COX-2 might participate in oncogenesis of NSCLC. Similar COX-2 positivity rates ranging from 54 to 100% have been reported for NSCLC tumors as measured by immunohistochemistry [34].

In our study it was found that COX-2 protein expression in adenocarcinoma was significantly higher than that in squamous carcinoma (*p *= 0.022), which was consistent to previous findings of other study [21]. This might provide basis for applying COX-2 inhibitor in adenocarinoma patients receiving tyrosine kinase inhibitor (TKIs), as COX-2 inhibitor offered synergistic antitumor effects with TKI [21].

Although COX-2 expression was also found higher in female patients, patients with ages≤60 years, non-smokers, moderate and well differentiated tumors, nodal metastasis, and in stages III-IV, the difference had no statistical significance. Studies examining the relationship between COX-2 tumor expression and survival among lung cancer patients were inconsistent, with reports of an inverse relationship with survival [35], no association [36], or a direct association with survival [37]. In our study, there was no correlation between COX-2 expression and patient's overall survival. However, unlike some previously reported studies which showed that COX-2 expression was most consistently associated with poorer survival among stage I and II NSCLC patients [38,39], our study neither showed the correlation of COX-2 expression with patient's survival nor prognostic value in early stage adenocarcinma [21]. This might be due to the small sample size in our study.

No correlation was found between EGFR expression and COX-2 in our study, though both EGFR and COX-2 involve in the carcinogenesis and progression of NSCLC both individually and, as recently suggested, synergistically [40]. A number of *in vitro *studies postulated a link between EGFR activation and subsequent COX-2 up-regulation. EGFR activation could induce COX-2 expression via the ras/raf MAPK pathway [3]. On the other hand, COX-2 could induce the activation and expression of EGFR. The lack of correlation of EGFR and COX-2 expression in our study implied that the expression of these 2 proteins might be controlled by independent mechanisms. As suggested by a recent study that examined the expression of p-EGFR, EGFR, and COX-2 by immunohistochemistry in surgically-resected stage I/II NSCLC, pathways other than EGFR activation may influence COX-2 overexpression[38]. Our results were similar: both EGFR and COX-2 are overexpressed in NSCLC; the predominant patterns of COX-2 and EGFR staining were cytoplasmic. However, in our study, the positivity of COX-2 in tumor was as high as 90%, and the number of cases was too small to analyze survival with further stratification between COX-2 and EGFR positive patients. It might be possible that the dual positive expression of COX-2 and EGFR could exert synergistic prognostic and predictive effect on NSCLC survival [31]. Besides, as TKI is becoming the treatment of choice in EGFR gene mutated advanced NSCLC patients, the role of COX-2 positivity on patient's response to TKI might be worthy of further investigation with larger samples. However, it was reported in recently published clinical trials that combined therapy with COX-2 inhibitors and the EGFR inhibitors had no additional benefit in patients who were not responsive to platinum therapy or who were chemotherapy-naive when compared to efficacy reported in previous studies with treatment of EGFR inhibitors alone [41,42]. Though there was no correlation between EGFR and COX-2 in NSCLC, they might remain as potential, though independent targets for treatment.

## Conclusions

In conclusion, this preliminary study illustrated that COX-2 and EGFR are both over-expressed in NSCLC. EGFR not only is an independent prognostic factor for overall survival but also a predictive factor for NSCLC receiving radiotherapy. The prognostic value of EGFR and COX-2 co-expression needs further study.

## Competing interests

We declare that we have no financial and personal relationships with other people or organizations that can inappropriately influence our work, and there is no professional or other personal interest of any nature or kind in any product, service and/or company that could be construed as influencing the position presented in, or the review of, the enclosed manuscript.

## Authors' contributions

FL carries out the design of the study and drafting the manuscript; HC is responsible for the control of pathological observation; YL worked on the analysis; DL and YS participated in the immunohistochemical process; FX and JW contribute equally to the conception of this study and the final approval of the version to be published. All authors read and approved the final manuscript.
