# Correction: EGFR and COX-2 protein expression in non-small cell lung cancer and the correlation with clinical features

**DOI:** 10.1186/1756-9966-30-32

**Published:** 2011-03-28

**Authors:** Feng Li, Yongmei Liu, Huijiao Chen, Dianying Liao, Yali Shen, Feng Xu, Jin Wang

**Affiliations:** 1 Radiation Oncology, Tumor Center, West China Hospital, Sichuan University, PR China; 2 Department of Pathology, West China Hospital, Sichuan University, PR China

## Correction

In the article [1] there were errors in Tables three, four, five, six and seven. The incorrect values were produced due to typographical errors during translation stage. These errors affect neither the published discussion nor the conclusions of the paper. However, a few changes to the results section are detailed here.

## In the Abstract, under "Results" the first two sentences read

"The positive rate of EGFR protein in NSCLC tumor cells was 46%, which was significantly higher than its expression in normal lung (p = 0.0234) and paracancerous tissues (p = 0.020). EGFR expression was significantly higher in nodal positive than in nodal negative patients (p = 0.04)."

But should have been:

"The positive rate of EGFR protein in NSCLC tumor cells was 46%, which was significantly higher than its expression in normal lung (p = 0.034) and paracancerous tissues (p = 0.020). EGFR expression was significantly higher in nodal positive than in nodal negative patients (p = 0.006)."

## In the main "Results" section of the article

The sentence under the heading "**EGFR protein expression**" read: "The positive rate of EGFR protein in NSCLC tumor cells were 46%, which was significantly higher than its expression in normal lung (p = 0.0234) and paracancerous (p = 0.020)"

Which should have been:

"The positive rate of EGFR protein in NSCLC tumor cells were 46%, which was significantly higher than its expression in normal lung (p = 0.034) and paracancerous (p = 0.020)"

Under the heading "**Correlation between EGFR expression and clinical features**" The second sentence read: "It shows that the difference of EGFR expression was only significant between the nodal positive and negative subgroups (56.4% vs.10%, p = 0.04)."

But the passage should have been "The expression of EGFR in different subgroups were compared and summarized in Table three. It shows that the difference of EGFR expression was only significant between the nodal positive and negative subgroups (56.4% vs. 9.1%, p = 0.006). There is no significant difference between age (60 vs. under 60 ys), gender, adeno- vs. non-adenocarcinoma, the differentiation of tumor, and staging."

This is the correct table three (table 1).

**Table 1:** (corrected table 3). EGFR expression and clinical characteristics

Clinical features	EGFR		Positive expression rate	*P *value
			
	negative	positive		
Ages				0.448
< 60	18	14	43.80%	
≥60	9	9	50%	
Sex				0.445
Male	16	15	48.40%	
Female	11	8	42.10%	
Pathologic type				0.543
Squamous carcinoma	13	8	38.10%	
Adencarcinoma	13	13	50.0%	
Mixed type	1	2	66.70%	
Tumor length				0.535
≤3 cm	9	7	43.80%	
> 3 cm	18	16	47.10%	
Level of Differentiation				0.474
Poor Differentiated	6	4	40%	
Moderate and Well Differentiated	21	19	47.50%	
TNM Stage				0.194
I-II	10	5	33.30%	
III-IV	17	18	51.40%	
Lymph node				0.006*
N0	10	1	9.10%	
N1-3	17	22	56.40%	

*P < 0.05

Correct tables four (table 2), five (table 3) and six (table 4).

**Table 2:** (corrected table four) COX-2 expression in neoplastic and normal tissue

Tissue type	Number of cases	COX-2		Positive rate(%)	P value
				
		positive	negative		
Neoplastic tissue	50	45	5	90	0.000*
Normal tissue	6	0	6	0	

P < 0.05

**Table 3:** (corrected table five) COX-2 expression in tumor and paracancerous tissue

Tissue type	Number of cases	COX-2		Positive rate(%)	P value
				
		positive	negative		
Neoplastic tissue	50	45	5	90	0.000*
Paracancerous tissue	7	1	6	14.3	

P < 0.05

**Table 4:** (corrected table six) 6 COX-2 expression and correlation with clinical features

Clinical features	COX-2		Positive expression rate	*P *value
			
	negative	positive		
Ages				0.599
≤60	3	30	90.90%	
> 60	2	15	88.20%	
Sex				0.362
Male	4	27	87.10%	
Female	1	18	94.70%	
Pathologic type				0.022*
Squamous carcinoma	5	16	76.20%	
Adencarcinoma	0	26	100%	
Mixed type	0	3	100%	
Tumor length				0.518
≤3 cm	2	14	87.50%	
> 3 cm	3	31	91.20%	
Level of Differentiation				0.258
Poor Differentiated	2	8	80%	
Moderate and Well Differentiated	3	37	92.50%	
TNM Stage				0.476
I-II	2	13	86.70%	
III-IV	3	32	91.40%	
Lymph node				0.699
N0	1	10	90.90%	
N1-3	4	35	89.70%	

*P < 0.05

Under the heading "**Correlation of EGFR and COX-2 expression**" The sentence reads: "As shown in Table seven, no correlation was found between COX-2 and EGFR protein expression (**Χ**2 = 0.112, P = 0.555)."

But should have read: "As shown in Table seven, no correlation was found between COX-2 and EGFR protein expression (P > 0.05)."

Correct table seven (Table 5).

**Table 5:** (corrected table seven) Correlation of EGFR and COX-2 protein expression

		EGFR		Total
			
		negative	Positive	
COX-2	negative	3	2	5
	positive	24	21	45
	Total	27	23	50

There was no significant relationship between COX-2 and EGFR. P > 0.05.
